# Deciphering *BAX* and *BCL2L12* circRNAs in Acute Myeloid Leukemia Through an Integrated Next-Generation and Nanopore Sequencing Approach

**DOI:** 10.3390/genes17080915

**Published:** 2026-08-01

**Authors:** Christina D. Sotiropoulou, Christos K. Kontos, Giannis Vatsellas, Vasiliki Pappa, Andreas Scorilas, Sotirios G. Papageorgiou

**Affiliations:** 1 Department of Biochemistry and Molecular Biology, Faculty of Biology, National and Kapodistrian University of Athens, Panepistimiopolis, 15771 Athens, Greece; xristswt@biol.uoa.gr (C.D.S.); ascorilas@biol.uoa.gr (A.S.); 2 Institute of Applied Molecular Biology—Biomarkers and Omics Technologies, University Center of Research and Innovation “Antonis Papadakis”, National and Kapodistrian University of Athens, 10561 Athens, Greece; 3 Institute of Molecular Typing of Hematological Neoplasms, University Center of Research and Innovation “Antonis Papadakis”, National and Kapodistrian University of Athens, 10561 Athens, Greece; vaspappa@med.uoa.gr (V.P.); sotpapage@med.uoa.gr (S.G.P.); 4 Greek Genome Center, Biomedical Research Foundation, Academy of Athens, 11527 Athens, Greece; gvatsellas@bioacademy.gr; 5 Second Department of Internal Medicine and Research Unit, University General Hospital “Attikon”, National and Kapodistrian University of Athens, 12462 Athens, Greece

**Keywords:** circular RNAs, circular transcripts, circRNA isoforms, alternative splicing, third-generation (long-read) sequencing, hematological malignancies

## Abstract

**Background:** Circular RNAs (circRNAs) constitute an emerging research field, as these RNA molecules play a crucial role in cellular functions and the progression of various human pathologies. Little is known about alternative circularization leading to the formation of distinct circRNAs from the same primary transcript, the role of circRNAs with slightly different back-splice junctions (BSJs) resulting in very similar circRNA sequences—called circRNA isoforms—and the extent to which the same primary transcripts produce alternative circRNAs. In this study, we discovered alternative circRNAs produced by two apoptosis-related genes, *BAX* and *BCL2L12*, expressed in established human cell lines originating from myelodysplastic syndrome (MDS) and different types of acute myeloid leukemia (AML). **Methods:** After total RNA extraction from one MDS cell line and five AML cell lines, first-strand cDNA synthesis, and multiple nested PCRs with distinct sets of divergent primers (10 and 16 primer pairs for *BAX* and *BCL2L12* circRNAs, respectively) annealing in each exon of *BAX* and *BCL2L12* genes, amplicon libraries were prepared and sequenced by both nanopore sequencing and NGS. Detailed bioinformatic analysis was then performed, based on existing bioinformatic tools and our own algorithms. **Results:** Our approach led to the identification of 72 *BAX* circRNAs and 52 *BCL2L12* circRNAs with distinct expression patterns in MDS and AML cell lines. Most of these circRNAs—either merely exonic or exonic–intronic—were detected for the very first time. Furthermore, several *BAX* circRNA isoforms were detected in a unique cell line. Moreover, the back-splice sites joined together to form the BSJ of each circRNA were non-canonical, in many cases. The identified circRNAs are predicted to sponge distinct sets of miRNAs, some of which are known to regulate the activity of pivotal pathways. **Conclusions:** Overall, our findings support the notion that alternative splicing and back-splicing lead to the production of tens of distinct circRNAs from the same human gene.

## 1. Introduction

Acute myeloid leukemia (AML) is an aggressive hematopoietic stem cell disorder characterized by the clonal overproduction of abnormal, poorly differentiated myeloid progenitor cells. These malignant transformations disrupt normal bone marrow function, leading to hematological failure [1]. AML progression is typically rapid and encompasses a highly heterogeneous array of molecular and cytogenetic subtypes, which dictate clinical prognosis and therapeutic strategies [2]. Currently, the International Consensus Classification (ICC) and the World Health Organization (WHO) classification systems serve as the dual frameworks for stratifying these AML subtypes based on recurrent genetic abnormalities [3,4]. Closely linked to AML is myelodysplastic syndrome (MDS), a pre-leukemic clonal disorder characterized by peripheral blood cytopenias, ineffective hematopoiesis, and a high propensity for transformation into AML [5]. Both malignancies share a complex molecular landscape driven by disrupted epigenetic modifications, histone alterations, aberrant pre-mRNA splicing, and altered transcription factor networks [6,7]. While the precise molecular choreography triggering the evolution of MDS to secondary AML remains incompletely understood, it is driven by a cumulative acquisition of cytogenetic and molecular aberrations [8].

A critical determinant in the pathogenesis and progression of both MDS and AML is the deregulation of the homeostatic balance between hematopoietic cell proliferation and programmed cell death [9]. Apoptosis, the primary mechanism of programmed cell death, is strictly governed by the BCL2 protein family via the intrinsic (mitochondrial) pathway [10,11]. Consequently, these proteins have emerged as vital prognostic biomarkers and therapeutic targets in hematological malignancies [12]. *BCL2L12*, a structurally distinct member of the BCL2 family, is implicated in different types of cancer, including leukemias and lymphomas, with remarkable transcriptional complexity. This gene has been shown to generate more than 50 alternatively spliced variants, the differential expression of which across malignant cell types suggests context-dependent functional roles in tumor cell survival [13,14]. Elevated *BCL2L12* expression is associated with inferior disease-free survival in AML patients [15], as well as shorter overall survival in chronic lymphocytic leukemia (CLL) [16]. *BCL2L12* mRNA expression is also a significant predictor of response of children with acute lymphoblastic leukemia to the Berlin-Frankfurt-Münster chemotherapy protocol and of their overall survival [17].

On the other hand, BAX functions as a core pro-apoptotic executioner within this family, and its dysregulation is widely documented across myeloid malignancies [18,19]. Paradoxically, high *BAX* mRNA expression has been associated with poor outcomes and a failure to achieve complete remission in certain AML cohorts, suggesting that its prognostic value may be tied to its intracellular localization and structural activation state [20,21]. Given these dynamics, the direct therapeutic activation of BAX represents a promising strategy in AML treatment [22].

Beyond conventional linear transcripts, circular RNAs (circRNAs) have emerged as crucial regulatory components of the eukaryotic transcriptome. Produced via the non-canonical back-splicing of primary transcripts, these single-stranded, covalently closed RNA loops lack 5′ and 3′ ends, rendering them highly resistant to degradation by RNA exonucleases [23]. Although most annotated circRNAs are exonic, they can also incorporate intronic or intergenic sequences. Functionally, circRNAs act as protein scaffolds, templates for micropeptide translation, and, most notably, competitive endogenous RNAs (“sponges”) that sequester microRNAs (miRNAs) and RNA-binding proteins (RBPs) [24]. In hematological malignancies, deregulated circRNAs contribute to oncogenesis by silencing tumor-suppressive miRNAs or stabilizing oncogenic networks [25]. Furthermore, recurrent chromosomal translocations characteristic of AML and MDS have been shown to directly generate novel fusion-circRNAs that actively drive leukemogenesis [26].

Despite their established regulatory significance, our understanding of the alternative splicing, diversity, and specific pathological roles of apoptosis-related circRNAs in myeloid malignancies remains limited. Short-read massive parallel sequencing often fails to fully resolve the complex internal structures of full-length circRNAs, necessitating integrated transcriptomic approaches. Driven by the pivotal roles of *BAX* and *BCL2L12* in the intrinsic apoptotic pathway, this study aimed to comprehensively map the circRNA landscape of these two key loci. Here, we implement a targeted, high-throughput approach combining the single-nucleotide accuracy of short-read, next-generation sequencing (NGS) with the structural resolution of third-generation Nanopore long-read sequencing. Through this integrated genomic strategy, we characterize the presence, structural diversity, and expression profiles of *BAX*- and *BCL2L12*-derived circRNAs across a panel of five AML cell lines and one MDS cell line, providing new insights into the non-coding landscape of myeloid disorders.

## 2. Materials and Methods

### 2.1. Cell Culture

One MDS cell line (MDS-L) and five AML cell lines (HL-60, NB-4, Kasumi-1, MOLM-13, and U-937) were used in the current study. The HL-60, Kasumi-1, and U-937 cell lines were purchased from the American Type Culture Collection (ATCC^®^) (Manassas, VA, USA). The NB-4 and MOLM-13 cell lines were purchased from the German Collection of Microorganisms and Cell Cultures GmbH (DSMZ) (Braunschweig, Germany). The MDS-L cell line was a kind gift from Professor Kaoru Tohyama [27]. All cell lines were propagated according to the standard guidelines of each supplier.

### 2.2. Total RNA Extraction and Reverse Transcription

Total RNA extraction from MDS and AML cell lines was carried out using the TRItidy G™ Reagent (AppliChem GmbH, Darmstadt, Germany). The purity and the concentration of each RNA sample were evaluated by spectrophotometry. Subsequently, 2 µg of each RNA sample was subjected to reverse transcription using the highly thermostable Maxima™ H Minus Reverse Transcriptase (Thermo Scientific™, Thermo Fisher Scientific Inc., Waltham, MA, USA), so as to avoid template switching during reverse transcription, and random hexamers (New England Biolabs Ltd., Hitchin, UK), following the manufacturer’s instructions.

### 2.3. Nested PCR Using Divergent Primers

To amplify circRNAs only during the polymerase chain reaction (PCR) assay, two pairs of divergent primers in each exon were designed for *BAX* and *BCL2L12* genes (Tables S1 and S2). The cDNAs from human cell lines were subjected to a first-round PCR using KAPA Taq DNA Polymerase (KAPA Biosystems Inc., Woburn, MA, USA) in a MiniAmp Thermal Cycler (Applied Biosystems™, Thermo Fisher Scientific Inc.). Due to the low circRNA levels, a second-round PCR (semi-nested or nested PCR) was then conducted. The PCR products from the first-step PCR were diluted at a ratio of 1:50 in nuclease-free H_2_O, and 1 μL of the diluted products was used in the mix for the semi-nested or nested PCR. The thermal protocol was conducted as follows: A denaturation step at 95 °C for 3 min, followed by a cycling step (25 cycles for *BAX* and 30 cycles for *BCL2L12*) for 1 min, a denaturation step at 95 °C for 30 s, an annealing step at varying temperature (Tables S1 and S2) for 30 s, and an elongation step at 72 °C for 1 min. A final elongation step was carried out at 72 °C for 1 min.

All nested and semi-nested PCR products were electrophorized on 2% agarose gels and visualized under UV light after staining with ethidium bromide. Afterward, the PCR products of each cell line were mixed and purified using spin columns (Macherey-Nagel GmbH & Co. KG, Düren, Germany). The final concentration of the purified products was evaluated using a Qubit 2.0 fluorometer (Invitrogen™, Thermo Fisher Scientific Inc.). Last but not least, capillary electrophoresis using a bio-fragment analyzer Qsep1 (BiOptic, Inc., New Taipei City, Taiwan) was followed to determine the average length of PCR products present in each mixture.

### 2.4. Targeted Nanopore Sequencing for Identification of BAΧ and BCL2L12 circRNAs

The PCR products from each cell line were purified and used to create a barcoded library for nanopore sequencing. First, the purified PCR products underwent end-repair and ligation with various barcodes. Then, sequencing adapters were ligated, resulting in the construction of 12 barcoded libraries. These libraries represented the *BAX* and *BCL2L12* circRNAs from different cell lines. Amplicon nanopore sequencing was performed using the Flongle adapter on a MinION Mk1C sequencer (Oxford Nanopore Technologies plc., Oxford, UK), following the manufacturer’s instructions.

The primary analysis of the acquired nanopore-sequencing data was performed using Guppy [28], which encompassed base calling to convert raw electrical signals into nucleotide sequences, followed by demultiplexing to assign individual reads to their respective barcoded sample. Adapter and barcoded sample sequences were subsequently trimmed to retain only the desired amplicon. The obtained raw nanopore sequencing data were mapped to chromosome 19 of the human reference genome (GRCh38) using the Minimap2 aligner, with the splice-aware preset parameter appropriate for long-read sequencing data [29]; all other parameters were set to default. SAMtools were then used to generate BAM files and their indexes [30]. Mapped nanopore sequencing reads were visualized with the Integrative Genomics Viewer (IGV) (version 2.19.7) [31]. This visualization facilitated the detection of single-exon circRNAs. For the detection of the multi-exonic circRNAs, a subsequent analysis was conducted using the ASDT algorithm [32]. Finally, the reads representing circRNAs were selected and analyzed using our *in-house*–developed algorithms, as previously described [33]. The novel circRNAs were visualized using BED files, generated using BEDtools [34].

### 2.5. Next-Generation Sequencing (NGS) for Validation of the Identified circRNAs

The amplicons of the barcoded pools were validated using NGS. Therefore, an equimolar mixture of all barcoded pools of amplicons was used for NGS library preparation and sequencing using the single-end 500-bp (SE500) chemistry in an Illumina MiSeq Sequencing System (Illumina Inc., San Diego, CA, USA).

The raw NGS data was subjected to initial quality control assessment using FastQC. After adapter trimming using Cutadapt and quality-based filtering of the raw reads, alignment to chromosome 19 of GRCh38 was performed using Minimap2. SAMtools were then used to generate BAM files and their indexes [30]. Mapped NGS reads were visualized again with the Integrative Genomics Viewer (IGV) [31]. Per-base sequencing depth and coverage across the *BAX* and *BCL2L12* genomic regions were computed using the SAMtools depth and coverage functions, respectively.

### 2.6. Bioinformatic Analysis for the Prediction of Regulatory circRNA–miRNA–mRNA Axes

Aiming to predict the regulatory potential of the identified circRNAs, their miRNA sponging activity was assessed with the custom prediction tool of miRDB [35]. The miRNAs with a prediction score of ≥80% were used for further analysis. mRNA targets of the aforementioned miRNAs were predicted again using miRDB. The potential effect of the circRNAs on three major signaling pathways related to acute myeloid leukemia according to KEGG pathway [36], by regulatory circRNA–miRNA–mRNA axes, was assessed by exploring the involvement of predicted mRNA targets in these pathways.

## 3. Results

### 3.1. Exon Structure and Characteristics of the Identified BAΧ and BCL2L12 circRNAs

In our study, 72 *BAX* circRNAs and 52 *BCL2L12* circRNAs were identified in the MDS and/or AML cell lines, some of which had already been identified in previous studies of our research group, including a CLL cell line [37], seven colorectal cancer (CRC) cell lines [33,38], eleven breast cancer (BrCa) cell lines and a non-malignant one [39], as summarized in Table S3. Furthermore, only few circRNAs deriving from *BAX* and *BCL2L12* genes are listed in public repositories, including CircAtlas 3.0, CIRCpedia v.2, and circBase [40,41,42]. In fact, only circ-BAX-80 as well as circ-BCL2L12-37, circ-BCL2L12-38, and circ-BCL2L12-39 appear in at least one of these three databases. All novel *BAX* and *BCL2L12* circRNA sequences have been deposited in GenBank^®^ of NCBI.

**Figure 1:**
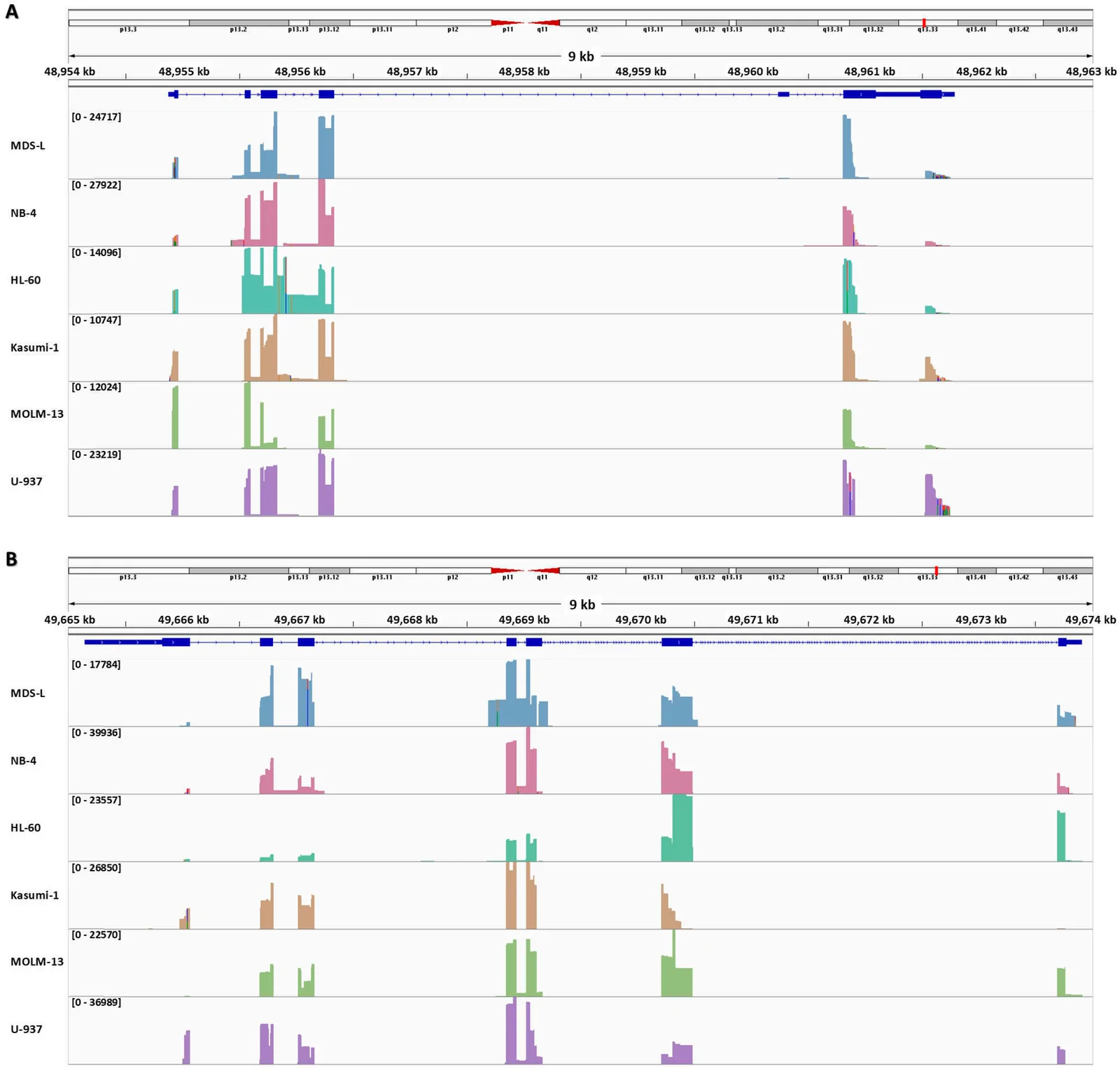
Visualization of the coverage of BAX (**A**) and BCL2L12 (**B**) genomic sequences by NGS reads of circRNA-derived amplicons in each cell line, using the Integrative Genomics Viewer (IGV). The scale of the vertical axis in all plots is linear. circRNAs cover a wider region of the primary transcript of each gene, compared to all mRNAs cumulatively (shown in dark blue).

The circRNAs of each gene cumulatively covered the already annotated exons (Figure 1), including *BAX* exon 5, which is present only in one nonsense-mediated mRNA decay (NMD) candidate. Moreover, some introns were retained in circular transcripts. This makes the cumulative coverage of circRNAs different from the one of mRNAs produced by each gene. Interestingly, the coverage of genomic *BAX* and *BCL2L12* regions was rather different among the 6 cell lines.

**Figure 2:**
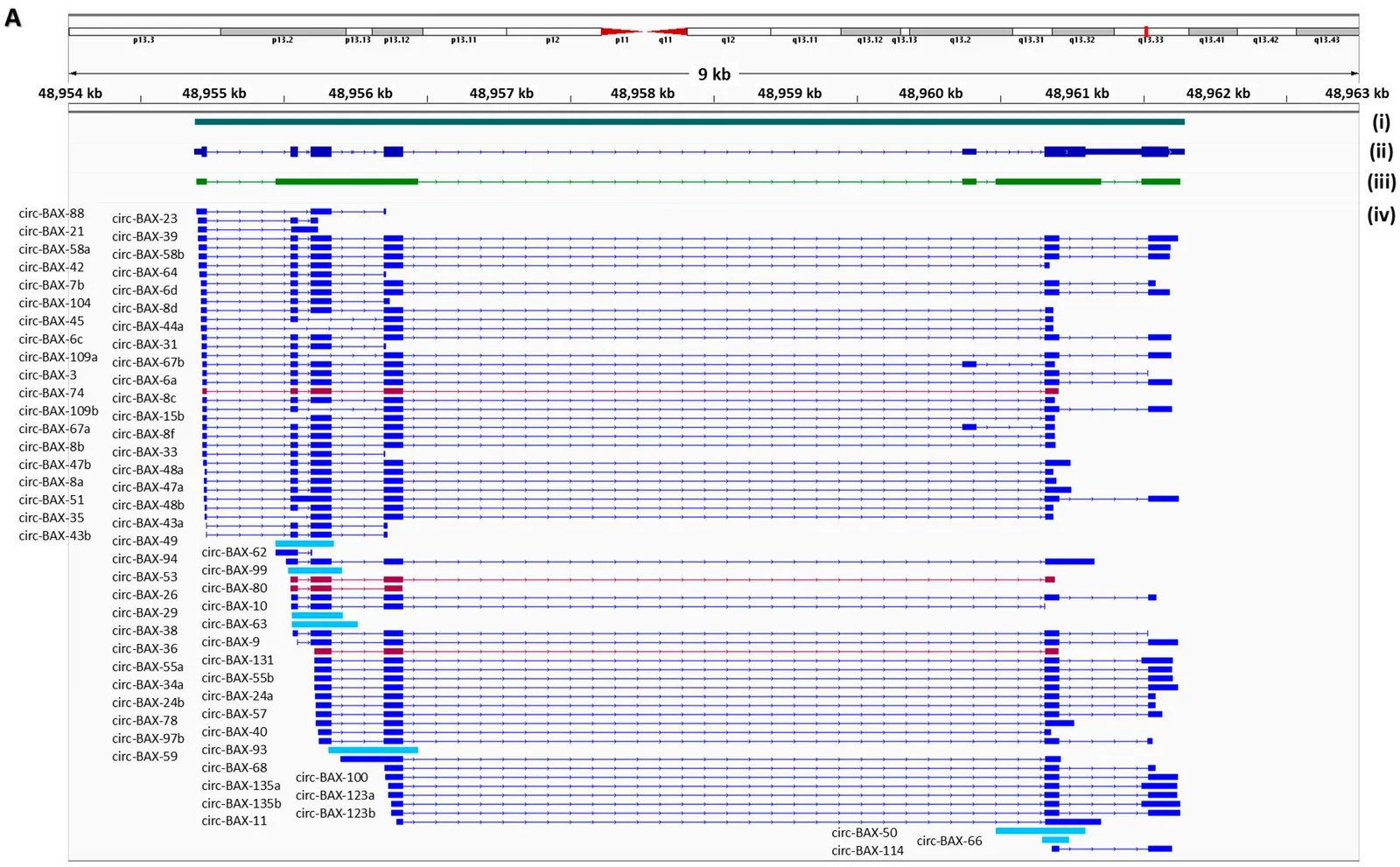

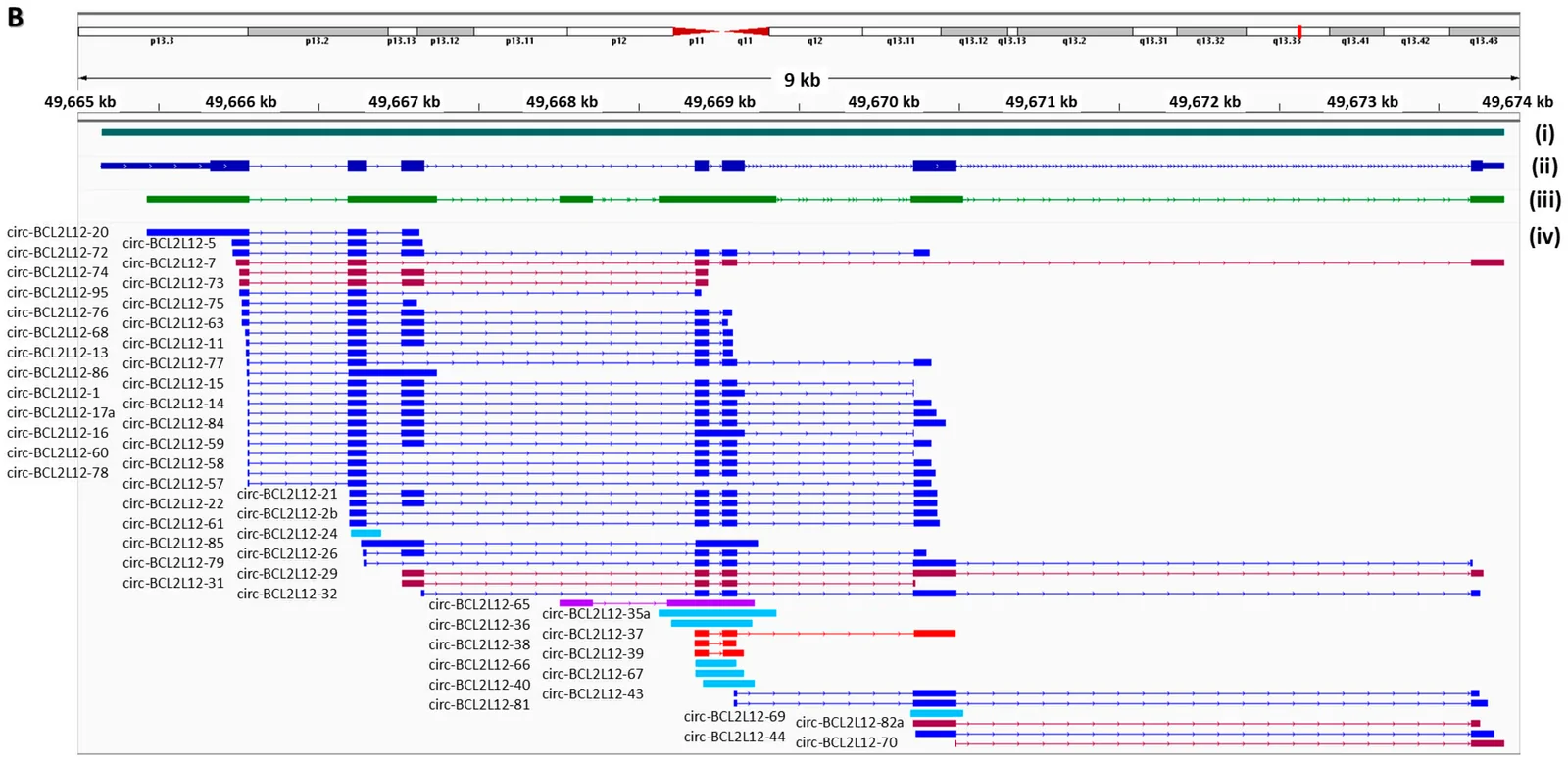
Representation of *BAX* (**A**) and *BCL2L12* (**B**) circRNAs in the 1 MDS and 5 AML cell lines, using the Integrative Genomics Viewer (IGV). (**A**) The alignment of circRNAs against chromosome 19 (chr19) of *Homo sapiens* genome assembly GRCh38.p14 (hg38) was done using Minimap2. The circRNAs cumulatively (**iii**) cover a much wider region of the primary transcript of this gene (**i**), compared to all mRNAs together (**ii**). The acceptor back-splice site of each circRNA constitutes the starting position of each aligned sequence (**iv**). Distinct colors of aligned sequences indicate subgroups—cyan: single-exon circRNAs; purple: circRNAs comprising one or two novel cryptic exons; jazzberry jam and red: circRNAs with one or both (respectively) back-spliced exons already known and of full length.

Regarding the exon structure of the novel circular transcripts (Figure 2), 7 *BAX* circRNAs and 7 *BCL2L12* circRNAs consisted of a single exon. All of them include at least one annotated exon plus part of the flanking introns, as their initial amplification was based on exon-specific primers. The single-exon circRNAs of each gene are highly overlapping, e.g., circ-BAX-49, circ-BAX-99, circ-BAX-29, circ-BAX-63, and circ-BAX-93 (Figure 2A), or circ-BCL2L12-35a, circ-BCL2L12-36, circ-BCL2L12-66, circ-BCL2L12-67, and circ-BCL2L12-40 (Figure 2B). Moreover, only 3 circRNAs produced by *BCL2L12* gene—namely circ-BCL2L12-37, circ-BCL2L12-38, and circ-BCL2L12-39—comprised only complete exons (Figure 2B), whereas no such *BAX* circRNAs were identified (Figure 2A). Interestingly, one circRNA (circ-BCL2L12-65) comprised an exon that had never been described before.

**Figure 3:**
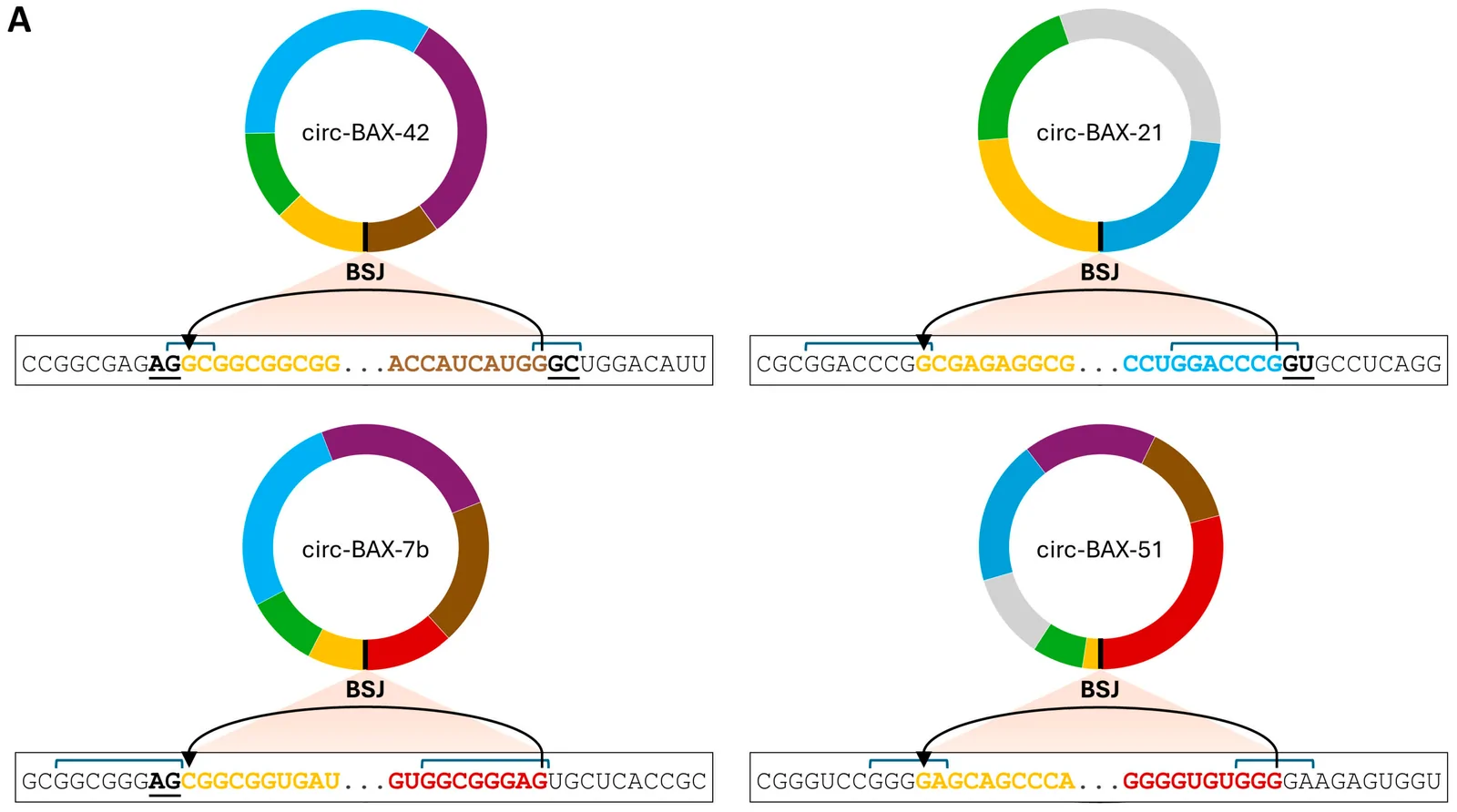

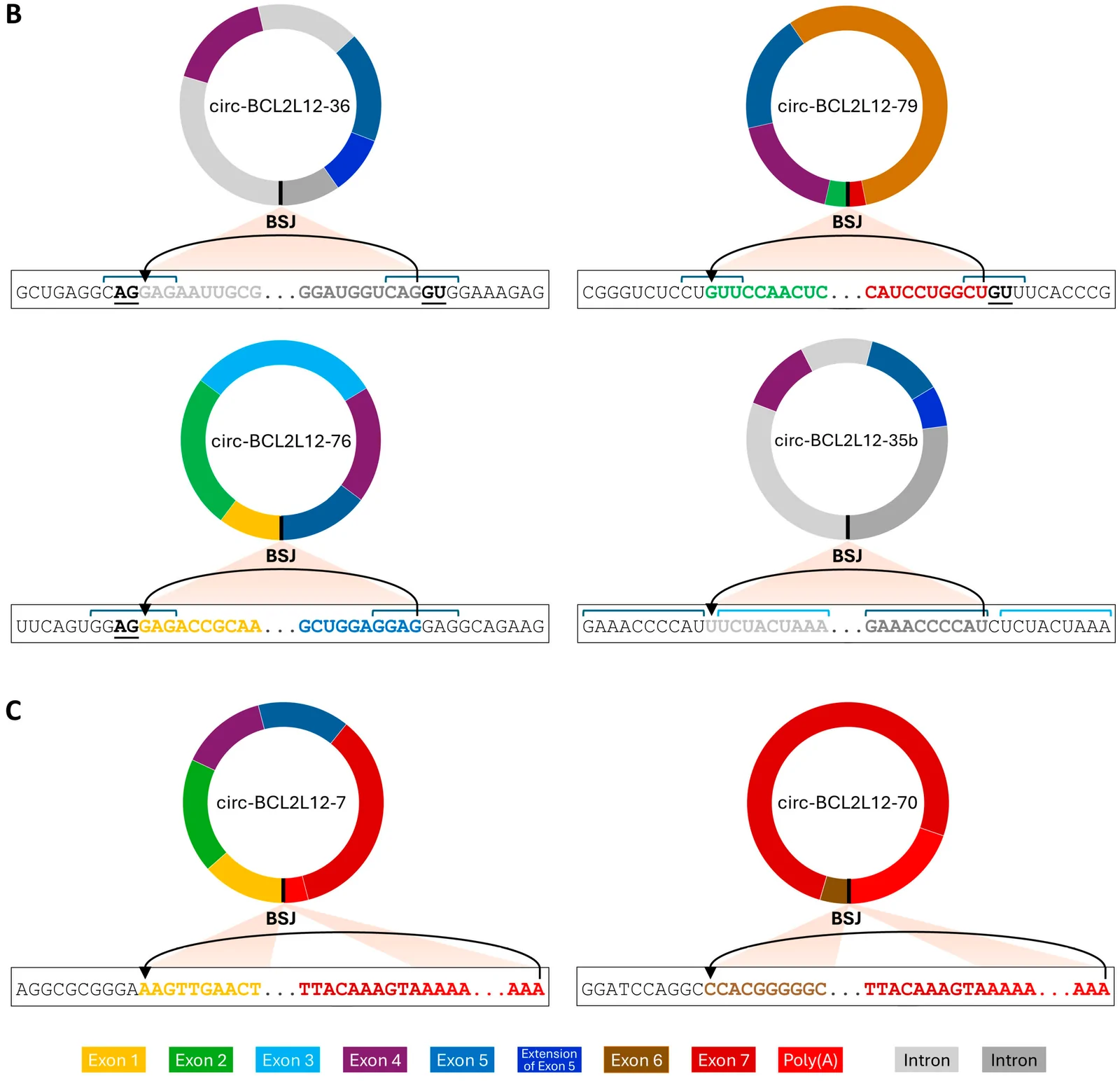
Schematic representation of selected circRNAs. Typical examples of *BAX* (**A**) and *BCL2L12* (**B**) circRNAs identified in the 6 cell lines, with both canonical back-splice sites (GU/AG or GC/AG), only one canonical back-splice site, and both non-canonical back-splice sites. Two of the latter contain a poly(A) tract (**C**), resulting from polyadenylation of the primary *BCL2L12* transcript as a step during its maturation, prior to its circularization. All exons and the poly(A) sequence are depicted as colored parts of the rings; introns are depicted as gray parts of the rings. Inside boxes containing sequences of the primary transcripts of each gene, exon ends (10 nucleotides) are written in colored bold font; “AAAA…AAA” written in red bold font indicates a poly(A) tract; intron boundaries (10 nucleotides) are written in standard black font; “…” indicates many other intervening nucleotides. All canonical back-splice sites are shown in black bold and underlined font. Horizontal square brackets over sequences indicate identical sequences of the primary transcripts of each gene. Arrows indicate BSJ formation. Abbreviation: BSJ, back-splice junction.

In general, among the identified *BAX* and *BCL2L12* circRNAs, the back-splice sites at the exon-intron boundaries being joined together to form the BSJ are rarely canonical (GU/AG or GC/AG). More precisely, circ-BAX-42 is the only one among the 72 *BAX* circRNAs possessing canonical back-splice sites (Figure 3A), whereas such *BCL2L12* circRNAs are 9 out of 52: circ-BCL2L12-24, circ-BCL2L12-36 (Figure 3B), circ-BCL2L12-37, circ-BCL2L12-38, circ-BCL2L12-39, circ-BCL2L12-66, circ-BCL2L12-67, circ-BCL2L12-68, and circ-BCL2L12-85. Moreover, 29 BAX circRNAs have either a canonical back-splice acceptor (AG) or donor (GT or GC), with circ-BAX-7b and circ-BAX-21 being such representative examples, illustrated in Figure 3A. The respective number of BCL2L12 circRNAs is 17, with circ-BCL2L12-76 and circ-BCL2L12-79 being such representative examples, illustrated in Figure 3B. Lastly, both back-splice sites of the majority of identified circRNAs (42 out of 72 *BAX* circRNAs and 26 out of 52 *BCL2L12* circRNAs) were non-canonical; circ-BAX-51 (Figure 3A) and circ-BCL2L12-35b (Figure 3B) constitute such representative examples.

Another interesting finding is that regions of the primary transcripts harboring non-canonical back-splice sites forming a BSJ are characterized by local sequence identity (usually 4–10 nucleotides). These identical sequences are located either in exons or in introns being back-spliced together. In fact, only one of such “pairs” of genomic regions is found in a circRNA sequence, while the other one is spliced out (Figure 3A,B).

circRNAs are considered circular transcripts lacking a 5′ cap and a poly(A) tail, namely two fundamental characteristics of the linear transcripts resulting from maturation of primary transcripts of RNA polymerase II. Surprisingly, a poly(A) tract following exon 7—the last exon of *BCL2L12* mRNAs—was found in 2 *BCL2L12* circRNAs (circ-BCL2L12-7 and circ-BCL2L12-70). Particularly, the back-splice junction (BSJ) of circ-BCL2L12-7 was formed between the poly(A) tract and a 5′-truncated exon 1, whereas the BSJ of BCL2L12-70 was formed between the poly(A) tract and a 5′-truncated exon 6 (Figure 3C).

### 3.2. Expression Profiling of BCL2L12 and BAX circRNAs in the MDS and AML Cell Lines

**Figure 4:**
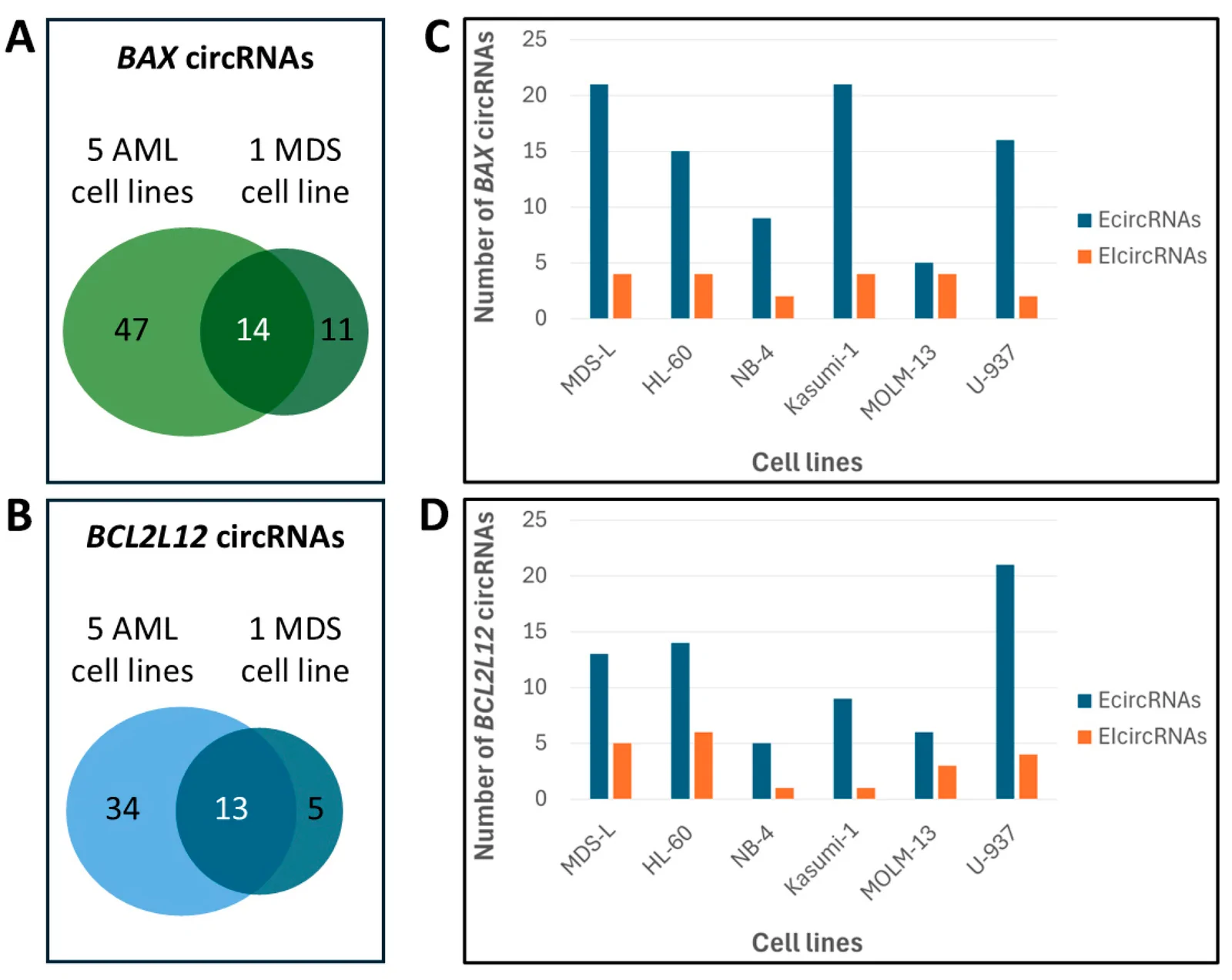
Comparative expression analysis of *BAX* and *BCL2L12* circRNAs in the single MDS cell line and the 5 AML cell lines. Venn diagrams show the unique and common circRNAs of *BAX* (**A**) and *BCL2L12* (**B**) genes between the single MDS cell line and the 5 AML cell lines. Bar plots show the numbers of exonic (EcircRNAs) and exonic–intronic (EΙcircRNAs) circRNAs of *BAX* (**C**) and *BCL2L12* (**D**) genes detected in the single MDS cell line and the 5 AML cell lines.

Our experimental approach based on nanopore sequencing also revealed the expression profile of *BAX* (Table S4) and *BCL2L12* circRNAs (Table S5) in the single MDS cell line and the 5 AML cell lines. Thus, out of the 72 *BAX* circRNAs detected in total, 61 transcripts are expressed in at least one AML cell line and 25 circular *BAX* transcripts are expressed in the MDS-L cell line, with 14 of them being common between MDS and AML cell lines (Figure 4A). Moreover, out of the 52 *BCL2L12* circRNAs detected in total, 47 transcripts are expressed in at least one AML cell line and 18 circular *BCL2L12* transcripts are expressed in the MDS-L cell line, with 13 of them being common between MDS and AML cell lines (Figure 4B). The expression profile of both genes differs among all studied cell lines (Tables S4 and S5); notably, only one BAX circRNA—namely circ-BAX-99—was detected in all cell lines studied.

The majority of circRNAs detected, produced either from *BAX* or *BCL2L12* gene, were exonic circRNAs (EcircRNAs), whereas only a few exonic–intronic circRNAs (EIcircRNAs) were identified (Table S6; Figure 4C and Figure 4D, respectively). Interestingly, EIcircRNAs of these two genes were detected in all six cell lines; therefore, it is evident that the identified circRNAs of *BAX* and *BCL2L12* cumulatively cover a larger portion of each gene and its primary transcript, compared to the previously annotated linear transcripts (Figure 2).

**Figure 5:**
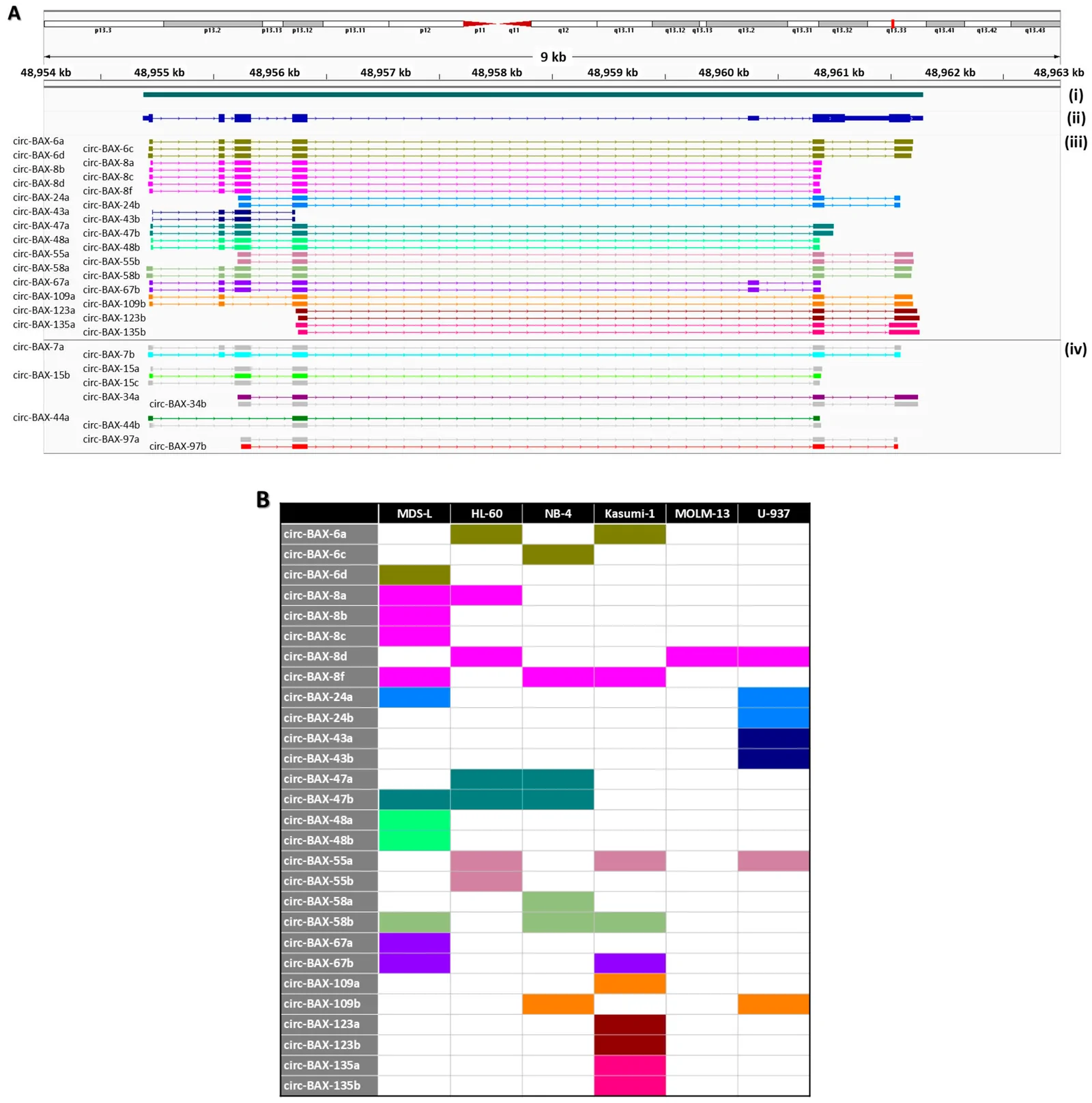
Exon structure comparison and expression analysis of *BAX* isoforms in the 1 MDS and 5 AML cell lines. (**A**) Visualization of the aligned *BAX* circRNA isoforms, using the Integrative Genomics Viewer (IGV). The acceptor back-splice site of each circRNA constitutes the starting position of each aligned isoform sequence (**iii** and **iv**); the primary *BAX* transcript of this gene (**i**) and all *BAX* mRNAs cumulatively (**ii**) are also shown, for comparison purposes. Each subgroup of *BAX* circRNA isoforms detected in MDS and/or AML cell line(s) is shown in a different color, whereas *BAX* circRNA isoforms previously identified in other cancer and/or leukemic cell lines are shown in grey (in the lower panel). (**B**) Expression of *BAX* circRNA isoforms discovered in MDS and/or AML cell line(s) is depicted using the same colors for circRNA isoforms as in (**A**).

Another interesting observation is that some *BAX* and *BCL2L12* circRNAs have the same exon composition, yet subtle differences are observed in their BSJ, as the exact back-splice sites may vary. These particular circRNAs are designated as circRNA isoforms; *BAX* circRNA isoforms are illustrated in Figure 5A (upper panel). Some *BAX* circRNA isoforms are co-expressed in the same cell line (Figure 5B), while others appear to be cell-line-specific. Interestingly, a few other *BAX* circRNAs can also be characterized as isoforms, considering that very similar circRNAs with slightly different BSJs (Figure 5A, lower panel) have previously been detected in other cancer cell lines [37,39]; the same is true for *BCL2L12* circRNA isoforms [33,37]. Therefore, the circRNA expression profile of these genes is unique for each cell line, compared also to cell lines originating from other malignancies.

### 3.3. Prediction of Targeted miRNAs and Their Impact on Protein-Coding Gene Expression

The identified circRNA sequences of both genes are predicted to bind several miRNAs (Tables S7 and S8), some of which are very likely to regulate the expression of key signaling proteins involved in major signaling pathways with deregulated activity in AML, namely Jak-STAT, MAPK, PI3K-Akt, mTOR, cell cycle and transcription. Such predicted *BAX* and *BCL2L12* circRNA–miRNA–mRNA axes are summarized in Table 1 and Table 2, respectively. Gene expression that could be indirectly influenced by the putative function of *BAX* and *BCL2L12* circRNAs as competing endogenous circRNAs sponging miRNAs involves members of the RAS superfamily, MAPKs, PI3K catalytic subunits, cyclins, transcription factors, and many others (Table 1 and Table 2).

## 4. Discussion

As a critical determinant of transcriptome and proteome diversity, alternative splicing is a major driver of regulatory complexity and functional versatility in eukaryotes [43]. Indeed, alternative splicing aberrations are increasingly recognized as pivotal events in cancer development and progression, representing promising biomarkers and therapeutic targets [44,45,46]. Recently, the research community has focused heavily on somatic mutations within the RNA-splicing machinery in clonal hematological disorders, such as myelodysplastic syndromes (MDS) and acute myeloid leukemia (AML) [47,48]. Specifically, a distinct AML subgroup characterized by mutations in genes encoding chromatin remodeling factors and/or spliceosome components has been associated with a particularly poor prognosis [49,50]. These findings support the hypothesis that aberrant alternative splicing is a fundamental driver of AML and MDS pathogenesis [51,52,53,54].

However, a branch of this pathway that remains largely unexplored involves circular RNA (circRNA) plurality as a result of alternative splicing and back-splicing. Emerging evidence is beginning to uncover the vital roles of circRNAs in various molecular and cellular processes [55,56]. Furthermore, accumulating data indicates that circRNAs regulate distinct steps in leukemogenesis, exhibiting highly cell-type-specific expression profiles during hematopoietic differentiation [25,57]. Consequently, elucidating circRNA dynamics holds significant potential for advancing our understanding of the molecular background of AML and MDS.

In the present study, we coupled nanopore sequencing with NGS to identify multiple *BAX* and *BCL2L12* circRNAs, characterized by highly diverse structural architectures. By combining these complementary sequencing technologies, we captured both novel splice junctions and intricate internal structural details with high precision and resolution. We also identified circRNAs produced via alternative back-splicing as well as isoforms sharing identical BSJs but showing different combinations of other exons. While these findings align with general trends in circRNA biology, they underscore the complex and highly regulated nature of splicing and back-splicing. Moreover, the circRNA expression profile of both genes was different among the six studied cell lines, as well as between each of these cell lines and any other human malignant cell line previously studied, including a CLL cell line [37] and several CRC and BrCa cell lines [33,38,39].

The structural analysis of the identified *BAX* and *BCL2L12* circular RNAs (circRNAs) reveals that their BSJs are predominantly formed at non-canonical exon-intron boundaries, with fully canonical splicing signals (GU/AG or GC/AG) being rather rare. While a minority of these transcripts possess a single canonical acceptor or donor site, the outright majority lack canonical sites at both ends of the BSJ. Interestingly, the genomic regions harboring these non-canonical sites are characterized by short, identical sequence stretches of 4 to 10 nucleotides located within the participating exons or introns; during back-splicing, only one copy of this duplicate sequence is retained in the mature circRNA, while the other is spliced out. Very recently, it has been suggested that among non-canonical splicing events, some are still tolerated and mediated by the U2/U12 spliceosome, whereas others appear to proceed through diverse and gene-specific mechanisms that remain to be further elucidated [58].

Furthermore, although circRNAs typically lack the classic hallmarks of linear mRNA transcripts, a unique structural variation was observed in two *BCL2L12* transcripts, in which a poly(A) tract following the final exon was unexpectedly retained and directly joined to a 5′-truncated upstream exon to form the BSJ. It should be noted, however, that this is not the first time that a poly(A) tract appears to constitute a part of a circRNA. Two other *BCL2L12* circRNAs comprising such a poly(A) tract, namely circ-BCL2L12-9 and circ-BCL2L12-55, have recently been discovered in CRC cell lines by members of our group; their sequences were independently confirmed by Sanger sequencing, too [33]. Moreover, these circRNAs were also present after RNase R treatment of the initial total RNA extracts isolated from those cell lines—a fact providing even stronger evidence of their existence rather than being experimental artefacts. In our study, all circRNA sequences—including those of circ-BCL2L12-7 and circ-BCL2L12-70—were discovered by nanopore sequencing and verified by means of NGS, thus enhancing the robustness of our findings.

The alternative processing of primary transcripts of *BAX* and *BCL2L12* genes yields a variety of linear transcripts encoding proteins with distinct, often opposing, functional properties. A similar functional diversity may apply to the intensive alternative splicing events observed among circular *BAX* and *BCL2L12* transcripts [59]. As the majority of the identified *BAX* and *BCL2L12* circRNAs were multi-exonic, their biogenesis may directly impact linear gene expression. Given the established competition model between canonical splicing and back-splicing [60], the generation of these multi-exonic circRNAs raises critical questions regarding their downstream effects on linear *BAX* transcript levels and subsequent protein expression. This hypothesis of splicing competition is further supported by the detection of unique splice sites exclusive to circRNAs, alongside the frequent retention of intronic regions within these circular structures [60]. Given the pivotal regulatory roles of the BCL2 family members in apoptosis, deciphering this splicing competition could reveal novel molecular mechanisms dictating cell death evasion in MDS and AML [61]. Undoubtedly, such hypotheses need to be supported by additional experimentation and resulting evidence.

Intriguingly, parts of *BAX* and *BCL2L12* genomic sequences considered as merely intronic have been shown to be incorporated into some circRNAs. Extensive intron retention is a highly noteworthy finding; previous studies suggest that intron-retaining circRNAs (EIciRNAs) are primarily localized in the nucleus, where they actively modulate the transcription of their parental genes [62]. Because dysregulated apoptosis is a hallmark of *de novo* AML development and progression of high-grade MDS to AML [8,63], the putative indirect regulation of linear apoptotic transcripts by these novel circRNAs—via miRNA sponging or formation competition—warrants deep investigation. Furthermore, recent studies have identified transcriptional shifts associated with hematopoietic differentiation during the transition from MDS to AML [7,64,65]. Thus, thorough understanding of how circRNAs may modulate the linear products of RNA splicing could provide insights into the molecular drivers of disease progression.

Crucially, many of the circRNA isoform profiles detected in our dataset represent structures not yet described in the literature. While the term “isoform” in circRNA biology typically refers to transcripts sharing a BSJ but differing in internal exon composition, the molecular mechanism generating this diversity remains poorly understood. The conservation of these specific isoforms across different AML and MDS cell lines suggests highly regulated expression patterns rather than stochastic splicing noise. Given that circRNAs are increasingly implicated in hematopoietic stem cell differentiation [66], these novel isoforms may reflect distinct cellular differentiation states and exert specialized regulatory functions during myelopoiesis.

Despite these insights, several limitations of this study must be acknowledged. First, our PCR-based enrichment and nested-PCR amplification workflow, while highly sensitive for novel circRNA discovery, is not suitable for accurate circRNA quantification. Consequently, our methodology is optimized for qualitative detection rather than precise abundance measurement. Second, while circRNAs hold great promise as clinical biomarkers and therapeutic targets in hematological malignancies, the functional roles of these novel *BAX* and *BCL2L12* circRNAs were not experimentally validated, in vitro.

Nonetheless, this study establishes a robust structural foundation for future functional and mechanistic research on circRNAs of particular genes. Future investigation should focus on validating these transcripts in MDS and AML patients’ samples and exploring the therapeutic potential of targeting specific circRNAs—either through direct RNA interference or by modulating their biogenesis—to restore apoptosis sensitivity in myeloid malignancies. Moreover, to understand whether the observed circRNA plurality is a hallmark of AML (or not), it would be very interesting to study the circRNA expression profile of *BAX* and *BCL2L12* in normal CD34+ cells and compare it with the profile documented in the single MDS and the 5 AML cell lines included in the current study.

## 5. Conclusions

In summary, we successfully identified 72 *BAX* and 52 *BCL2L12* circRNAs characterized by distinct expression patterns across MDS and AML cell lines. The majority of these molecules—being either exonic or exonic–intronic—are reported here for the first time, with several *BAX* isoforms exhibiting cell-line-specific expression. Notably, a significant proportion of the identified circRNAs are formed through back-splicing of non-canonical splice sites. Taken together, our findings support the notion that alternative splicing and back-splicing account for the generation of tens of distinct circRNAs from the same human gene. Furthermore, the prevalence of non-canonical BSJs challenges standard assumptions regarding RNA circularization requirements, suggesting the existence of alternative mechanisms that operate independently of canonical back-splicing signals.

## Figures and Tables

**Table 1:** Predicted target genes and associated KEGG pathways of miRNAs regulated by BAX circRNAs.

miRNA	Target Gene	KEGG Pathway
miR-152-5p	*PIK3CD*	PI3K-Akt signaling pathway
	*RPS6KB1*	mTOR signaling pathway
miR-1843	*CCNA1*	Cell cycle
miR-3150b-3p	*AKT3*	PI3K-Akt signaling pathway
	*NRAS*, *MAPK1*	MAPK signaling pathway
	*RARA*, *RUNX1T1*	Transcription
miR-3202	*BAD*, *EIF4EBP1*	PI3K-Akt signaling pathway
	*GRB2*	MAPK signaling pathway
	*PML*	Transcription
	*CCND1*, *PPARD*	Cell cycle
miR-3619-5p	*IKBKB*, *PIK3CB*	PI3K-Akt signaling pathway
	*PIM1*	JAK-STAT signaling pathway
	*MAPK1*, *MAPK3*, *SOS2*	MAPK signaling pathway
	*RUNX1*	Transcription
miR-4784	*AKT3*	PI3K-Akt signaling pathway
	*MAPK1*, *NRAS*	MAPK signaling pathway
	*RARA*, *RUNX1T1*	Transcription
miR-4802-5p	*CCNA1*	Transcription
miR-5590-5p	*RUNX1*	Transcription
miR-6529-3p	*HRAS*	MAPK signaling pathway
	*LEF1*	Cell cycle
miR-6729-3p	*ARAF*	MAPK signaling pathway
	*NFKB1*	PI3K-Akt signaling pathway
miR-6889-3p	*HRAS*	MAPK signaling pathway
	*LEF1*	Cell cycle
miR-9851-5p	*RPS6KB1*	mTOR signaling pathway
	*BRAF*, *SOS1*	MAPK signaling pathway
	*RUNX1T1*	Transcription

**Table 2:** Predicted target genes and associated KEGG pathways of miRNAs regulated by *BCL2L12* circRNAs.

miRNA	Target Gene	KEGG Pathway
miR-182-5p	*CEBPA*	Transcription
	*GRB2*, *SOS1*	MAPK signaling pathway
	*PIK3CA*, *PIK3R1*	PI3K-Akt signaling pathway
	*TCF7L2*	Cell cycle
miR-371b-5p	*NRAS*	MAPK signaling pathway
	*PIK3CA*, *PIK3CD*	PI3K-Akt signaling pathway
	*TCF7*	Cell cycle
	*RUNX1*	Transcription
miR-373-5p	*NRAS*	MAPK signaling pathway
	*PIK3CA*, *PIK3CD*	PI3K-Akt signaling pathway
	*TCF7*	Cell cycle
	*RUNX1*	Transcription
miR-516-b-5p	*JUP*	Cell cycle
	*PIK3CA*, *PIK3CB*, *PIK3R2*	PI3K-Akt signaling pathway
	*STAT3*	JAK-STAT signaling pathway
	*TCF7L2*	Cell cycle
	*CCNA2*	Transcription
miR-616-5p	*NRAS*	MAPK signaling pathway
	*PIK3CA*, *PIK3CD*	PI3K-Akt signaling pathway
	*TCF7*	Cell cycle
	*RUNX1*	Transcription
miR-708-3p	*SOS2*	MAPK signaling pathway
miR-1321	*AKT3*, *KIT*	PI3K-Akt signaling pathway
	*KIT*	MAPK signaling pathway
		JAK-STAT signaling pathway
	*PML*, *RUNX1T1*	Transcription
	*PPARD*	Cell cycle
miR-1915-5p	*AKT3*	PI3K-Akt signaling pathway
	*LEF1*	Cell cycle
miR-3613-3p	*RPS6KB1*	PI3K-Akt signaling pathway
	*RUNX1*	Transcription
miR-4739	*AKT3*, *KIT*	PI3K-Akt signaling pathway
	*KIT*	MAPK signaling pathway
		JAK-STAT signaling pathway
	*PML*, *RUNX1T1*	Transcription
	*PPARD*	Cell cycle
miR-4756-5p	*AKT3*, *KIT*	PI3K-Akt signaling pathway
	*KIT*	MAPK signaling pathway
		JAK-STAT signaling pathway
	*PML*, *RUNX1T1*	Transcription
	*PPARD*	Cell cycle
miR-7844-5p	*SOS2*	MAPK signaling pathway
	*PIK3R3*	PI3K-Akt signaling pathway

## Data Availability

The raw nanopore sequencing reads have been deposited to the Sequence Read Archive (SRA) of NCBI. All other datasets generated and/or analyzed during the current study are available from the corresponding author upon reasonable request.

## Supplementary Materials

The following supporting information can be downloaded at: https://www.mdpi.com/article/10.3390/genes17080915/s1. Table S1: First- and second-round PCR primer pairs, used to generate *BAX*-derived amplicons for the nanopore sequencing libraries; Table S2: First- and second-round PCR primer pairs, used to generate *BCL2L12*-derived amplicons for the nanopore sequencing libraries; Table S3: Newly vs. previously identified *BAX* and *BCL2L12* circRNAs; Table S4: Expression analysis of the identified *BAX* circRNAs in MDS and AML cell lines; Table S5: Expression analysis of the identified *BCL2L12* circRNAs in MDS and AML cell lines; Table S6: Types of the identified circRNAs in MDS and AML cell lines; Table S7: The miRNAs that are predicted to be sponged by each *BAX* circRNA, with the relative prediction score from custom prediction analysis with miRDB; Table S8: The miRNAs that are predicted to be sponged by each *BCL2L12* circRNA, with the relative prediction score from custom prediction analysis with miRDB.
